# Anti-CD40-induced inflammatory E-cadherin+ dendritic cells enhance T cell responses and antitumour immunity in murine Lewis lung carcinoma

**DOI:** 10.1186/s13046-015-0126-9

**Published:** 2015-02-05

**Authors:** Yong Zhang, Xiaoyan Hu, Yue Hu, Kai Teng, Kai Zhang, Yamei Zheng, Xiaohua Hong, Kunwu Yu, Yan Wang, Li Liu

**Affiliations:** Cancer Center, Union Hospital, Tongji Medical College of Huazhong University of Science and Technology, 156 Wujiadun, Wuhan, 430023 Hubei China; Hainan Cancer Hospital, Haikou, Hainan China; Department of oncology, Central Hospital of Wuhan, Wuhan, Hubei China; Laboratory of Cardiovascular Immunology, Institute of Cardiology, Union Hospital, Tongji Medical College of Huazhong University of Science and Technology, Wuhan, Hubei China; Institute of Hydro Biololgy, Chinese Academy of Sciences, Analysis and Testing center, Wuhan, China

**Keywords:** E-cadherin, Dendritic cell, T cell, Lung cancer, Activity

## Abstract

**Background:**

Agonistic CD40 antibodies have been demonstrated to activate antigen-presenting cells (APCs) and enhance antitumour T cell responses, thereby providing a new therapeutic option in cancer immunotherapy. In agonistic CD40 antibody-mediated inflammatory responses, a novel subset of E-cadherin + dendritic cells (DCs) has been identified, and little is known about the role of these DCs in tumour immunity. This study investigated the effect of anti-CD40-mediated inflammatory E-cadherin + DCs in murine Lewis lung carcinoma (LLC).

**Methods:**

The phenotype and characteristics of anti-CD40-mediated inflammatory E-cadherin + DCs isolated from the anti-CD40 model were assessed in vitro. The antitumour activity of E-cadherin + DCs were evaluated in vivo by promoting the differentiation of effector CD4+ T cells, CEA-specific CD8+ T cells and CD103+ CD8+ T cells and assessing their resistance to tumour challenge, including variations in tumour volume and survival curves.

**Results:**

Here, we demonstrated that anti-CD40-mediated E-cadherin + inflammatory DCs accumulate in the lungs of Rag1 KO mice and were able to stimulate naïve CD4+ T cells to induce Th1 and Th17 cell differentiation and polarisation and to inhibit regulatory T cell and Th2 responses. Importantly, with the adoptive transfer of E-cadherin + DCs into the Lewis lung cancer model, the inflammatory DCs increased the Th1 and Th17 cell responses and reduced the Treg cell and Th2 responses. Interestingly, following the injection of inflammatory E-cadherin + DCs, the CD103+ CD8+ T cell and CEA-specific CD8+ T cell responses increased and exhibited potent antitumour immunity.

**Conclusions:**

These findings indicate that anti-CD40-induced E-cadherin + DCs enhance T cell responses and antitumour activity in non-small cell lung cancer (NSCLC)-bearing mice and may be used to enhance the efficacy of DC-based peptide vaccines against NSCLC.

**Electronic supplementary material:**

The online version of this article (doi:10.1186/s13046-015-0126-9) contains supplementary material, which is available to authorized users.

## Introduction

CD40 is a tumour necrosis factor receptor superfamily member that is expressed on antigen-presenting cells (APCs) such as dendritic cells (DC), B cells, monocytes and some tumour cells. Recently, agonistic CD40 antibodies were applied in clinical trials targeting advanced pancreatic ductal adenocarcinoma (CP-870,893) and diffuse large B cell lymphoma (dacetuzumab and Chi Lob 7/4). The CD40 agonistic antibody has displayed excellent antitumour activity in the patients in these trials [1,2]. Many subsets of DCs exist in the agonistic CD40 antibody-mediated tumour microenvironment or under sterile inflammatory response conditions. However, the mechanism and function of CD40-mediated inflammatory DCs in cancer immunity are unknown.

In CD40 agonistic antibody-mediated inflammatory responses, a novel subset of E-cadherin + DCs has been identified. Although CD40 signalling is critical for the differentiation of inflammatory monocytes into E-cadherin + inflammatory DCs and the promotion of anti-CD40-mediated colitis has been confirmed in Rag1 KO mice [3], little is known regarding the role of E-cadherin + inflammatory DCs in tumour immunity.

Precisely how inflammatory DCs with tumour antigen peptides can induce a T cell response in tumour immunity is poorly understood. Here, we identified the inflammatory E-cadherin + DCs that accumulate in the lung during the anti-CD40 antibody-mediated inflammatory response. The phenotypes of these DCs are the same as those of spleen-derived inflammatory E-cadherin + DCs that are present during anti-CD40-mediated colitis. The agonistic CD40 mAb has not been universally accepted as a novel cancer therapy. Concerns include cytokine release syndromes, autoimmune reactions [4], thromboembolic syndromes, hyperimmune stimulation leading to activation-induced cell apoptosis or tolerance [5,6] and tumour angiogenesis, possibly as a result of the CD40-dependent activation of tumour endothelial cells [7]. These effects may cause unacceptable toxicity or promote tumour growth [8]. This study aimed to investigate the effects of anti-CD40-induced E-cadherin + DCs on the T cell response and antitumour activity in the tumour microenvironment. We found that inflammatory E-cadherin + DCs were present only in anti-CD40-mediated innate immunity, not innate, adoptive and tumour immunity. Our study will address the disadvantages of agonistic CD40 mAb in tumour therapy and may provide novel therapeutic strategies, as well as explain the pathogenesis of non-small cell lung cancer (NSCLC).

## Materials and methods

Additional materials and methods can be found in the Additional file 1.

### Animals

We obtained 6- to 8-week-old C57BL/6 mice from the Wuhan University Centre for Animal Experiments. B6.129S7-Rag1^tmiMom^/JNju (Rag1^−^/^−^) mice (background: C57BL/6) were provided by the Mode Animal Research Centre of Nanjing University. These Rag1^−^/^−^ mice were housed and maintained in individual ventilated cages (IVC) under specific pathogen-free conditions; C57BL/6 mice were housed in specific pathogen-free conditions but not under IVC conditions. All breeding was conducted in the Huazhong University of Science and Technology Centre for Animal Experiments according to the National Institutes of Health Guide for the Care and Use of Laboratory Animals.

### Cell isolation

Spleens were digested with collagenase VIII (Sigma) as previously described [9] and filtered using a 70-μm cell strainer (BD Biosciences) to obtain mononuclear spleen cells. Lung or lung tumour tissues were cut into pieces, and mononuclear cells were digested with collagenase V (Sigma) for 2 h at 37°C as described previously [10]. The resulting spleen or lung cell suspensions from anti-CD40 model mice were stained using E-cadherin, CD11c, CD4, CD103 and 7-AAD. The cells were first sorted for 7-AAD^−^CD11c^+^ cells using a FACS AriaIII sorter (BD Biosciences); 7-AAD + CD11c- cells were discarded. The CD11c + cells were then sorted into E-cadherin + and E-cadherin- DCs (E-cadherin+/− CD11c^high^ CD4-CD103-7-AAD-, purity >98%), as described (Additional file 1: Figure S1). The DC subsets were cultured in DMEM (GIBCO, Invitrogen) supplemented with 10% foetal bovine serum (FBS, GIBCO, Invitrogen), LPS (1 μg/ml), streptomycin (100 μg/ml) and penicillin (100 U/ml) [3].

Naive CD4+ T cells (CD4 + CD62L + CD44^low^) and naive CD8+ T cells (CD8 + CD62L + CD44^low^) were prepared from cell suspensions isolated from the spleens of 6-8-week-old C57BL/6 mice. The cells were isolated using a FACSAriaIII sorter. The cells were first sorted for CD4+ T cells, which were then sorted for CD62L + CD44^low^ cells. Similarly, CD8 + CD62L + CD44^low^ cells were sorted from the CD8+ T cells, and naive CD8+ T cells were obtained. The naive T cell (CD4+/CD8+ T) purities exceeded 99% (Additional file 1: Figure S2).

### T cell differentiation and polarisation assay

Cell suspensions were prepared from the spleens of the anti-CD40 model of Rag1^−/−^ or C57BL/6 mice. Briefly, 3.2 × 10^4^ E-cadherin + or E-cadherin- CD11c^high^CD4-CD103-7-AAD-cells (E-cadherin + or E-cadherin- DCs, respectively) were cultured with or without 5 μg/ml CEA421-435 peptide (Invitrogen) for 24 h; 3.5 × 10^5^ sorted naive CD4+ T cells were then added to the E-cadherin + or E-cadherin- DCs in complete RPMI 1640 medium supplemented with 10% FBS, IL-2 (100 IU/ml, R&D Systems), plate-bound anti-CD3 and soluble anti-CD28 (5 μg/ml each, eBioscience) [11], either under Th1, Th2 and Th17 conditions for 96 h or Treg conditions for 5 days. Then, the Th1, Th2 and Th17 cells and the relevant cytokines were analysed at 72 h and 96 h. Treg cells and the relevant cytokines were analysed at 72 h and on day 5. Cell supernatants were collected and stored at −80°C for subsequent analysis of CD4+ T cell differentiation using the mouse Th1/Th2/Th17/Th22 13-plex kit (eBioscience) and the mouse TGF-β1 simplex kit (eBioscience). For Th1, Th2 and Th17 effector T cell polarisation analysis, multiplying system cells were collected on day 3 and analysed with the mouse Th1/Th2/Th17 phenotyping kit (BD Biosciences); for the Treg cell analysis, the mouse Th17/Treg phenotyping kit (BD Biosciences) was used after multiplying system cells collected at day 5.

For CD8+ T cell differentiation analysis, 2.3 × 10^4^ E-cadherin + or E-cadherin- DCs that were cultured with 5 μg/ml CEA526-533 peptide (Invitrogen) for 24 h were cultured with 3 × 10^5^ sorted naive CD8+ T cells, anti-CD3 (1 μg/ml) and anti-CD28 (5 μg/ml). The naive CD8 + Tcells cultured with anti-CD3 (1 μg/ml) and anti-CD28 (5 μg/ml) (no DCs) were considered as a control. At 48 h, the supernatant was collected to detect IFN-γ secretion using the mouse IFN-γ ELISA Kit (R&D systems).

### In vivo experiments

For the in vivo experiments, 5 × 10^5^ E-cadherin + CD11c^high^CD103^−^CD4-7AAD- DCs from the spleens of the Rag1^−/−^ anti-CD40 mouse model were transferred into the orthotopic or subcutaneous lung tumour model by tail vein injection at day 7, day 14 or day 21 after the tumour model was established (the day of tumour cell injection was day 0). The same number of E-cadherin-CD11c^high^CD103-CD4-7AAD- DCs and 200 μl of PBS were injected as controls. The E-cadherin + and E-cadherin- CD11c^high^ cell fractions were cultured with 5 μg/ml CEA526-533 peptide (Invitrogen) for 24 h prior to i.v. injection into the LLC tumour-bearing mice. At day 28, the orthotopic lung tumour-bearing mice were sacrificed, and the tumour tissues were harvested and prepared to investigate the effects of the E-cadherin + DCs on the helper T cell response using PCR and western blotting analyses. Lung tissue from orthotopic tumour-bearing mice was also prepared as a cell suspension to assay CD103 expression in CD8+ T cells. Spleen lymphocytes of orthotopic tumour-bearing mice were stained for tetramer staining.

### Statistical analyses

All data are expressed as the mean ± SD. Significant results were assessed using analysis of variance. Statistical significance between alues was determined using Student’s t test, and a statistically significant difference between two test groups was defined as P < 0.05.

## Results

### The phenotype of inflammatory E-cadherin + DCs in the lung

**Figure 1:**
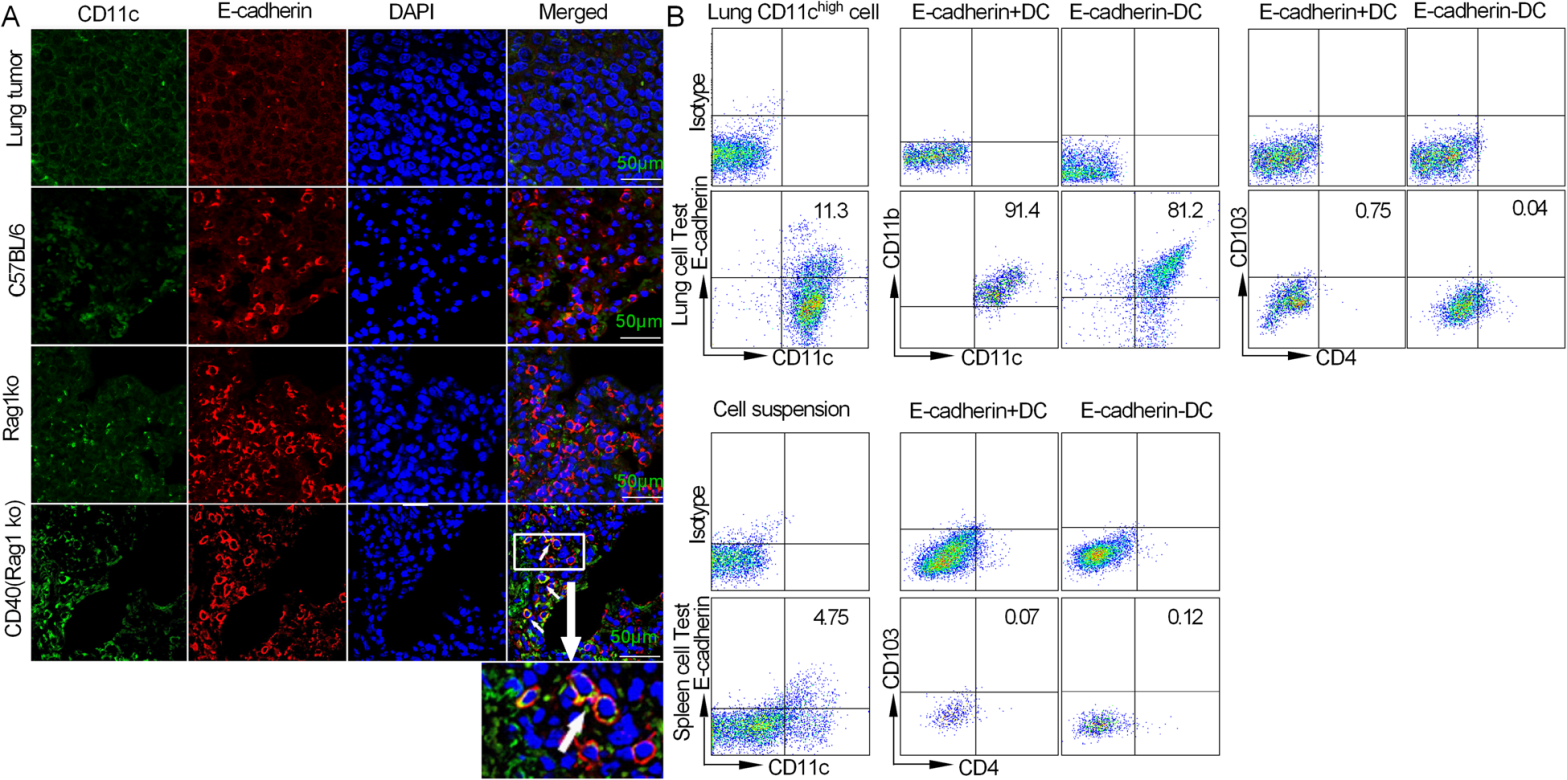
**E-cadherin expression on lung dendritic cell in terms of lung tumour immunity, adoptive immunity (C57BL6), innate immunity (Rag1 KO) and CD40-mediated immune response (CD40/Rag1 ko). (A)** Lung tissues were harvested from the Lewis lung cancer model, C57/BL6 mice, Rag1 KO mice and the anti-CD40 model, respectively. The green fluorescence represents CD11c, and red represents E-cadherin. **(B)** The phenotype of inflammatory E-cadherin + DCs in lung and spleen cell suspensions. The spleen and lung cells were harvested from the anti-CD40 model. For the lung cell test, E-cadherin and CD11c expression was analysed in sorted CD11c^high^ cells prepared from a lung single-cell suspension (sorted using a BD FACSAria III flow cytometer). Staining for CD11b, CD4, CD103 and E-cadherin and were gated on E-cadherin+/−CD11c^high^ cells from lung cells. For the spleen cell test, E-cadherin and CD11c expression was examined in a spleen single-cell suspension. Staining for CD4, CD103 and E-cadherin gated on E-cadherin+/−CD11c^high^ cells to analyse the expression of CD103 and CD4. Each experiment was performed in triplicate.

A subset of E-cadherin + inflammatory DCs has been identified in an anti-CD40 colitis model [3]. However, the presence of these CD40-mediated E-cadherin + DCs in the lung and their phenotype are unknown. To investigate the expression of E-cadherin in lung dendritic cells in the presence of different immune responses, we harvested lung tissue from the Lewis orthotopic lung cancer model, C57BL/6, Rag1 KO mice and Rag1 KO mice exposed to an anti-CD40 antibody, respectively. Interestingly, the fluorescence levels of E-cadherin and CD11c in the lung tumour were low, with E-cadherin only expressed in a minor fraction of the lung tissue of C57BL/6 mice, whereas the expression of E-cadherin and CD11c in the lung tissue of Rag1 KO mice exposed to an anti-CD40 antibody was increased and significantly greater than their expression in the Lewis orthotopic lung cancer model and C57BL/6 mice (Figure 1A). We then analysed whether E-cadherin was expressed in lung DCs undergoing a CD40 mAb-mediated inflammatory response. We showed that a minor subset of CD11c^high^ cells expressed E-cadherin in the lung, and approximately 11.3% of CD11c^high^ cells expressed E-cadherin during the CD40 mAb-mediated inflammatory response. The majority of lung-derived E-cadherin + and E-cadherin- DCs expressed high levels of CD11b and CD103 while expressing CD4 at low levels. The majority of E-cadherin + DCs expressed CD103 under steady-state conditions [3], whereas the phenotype of E-cadherin + DCs in the lung under anti-CD40 conditions was the same as that of E-cadherin + DCs in the spleen (E-cadherin + CD11c^high^CD11b^high^CD103-CD4-) (Figure 1B). These findings suggest that the phenotype of lung inflammatory E-cadherin + DCs mimics that of E-cadherin + DCs in the spleen [3] with regard to CD40 mAb-mediated inflammatory responses.

### The effects of E-cadherin + DCs on effector T cells (Th1 and Th2)

**Figure 2:**
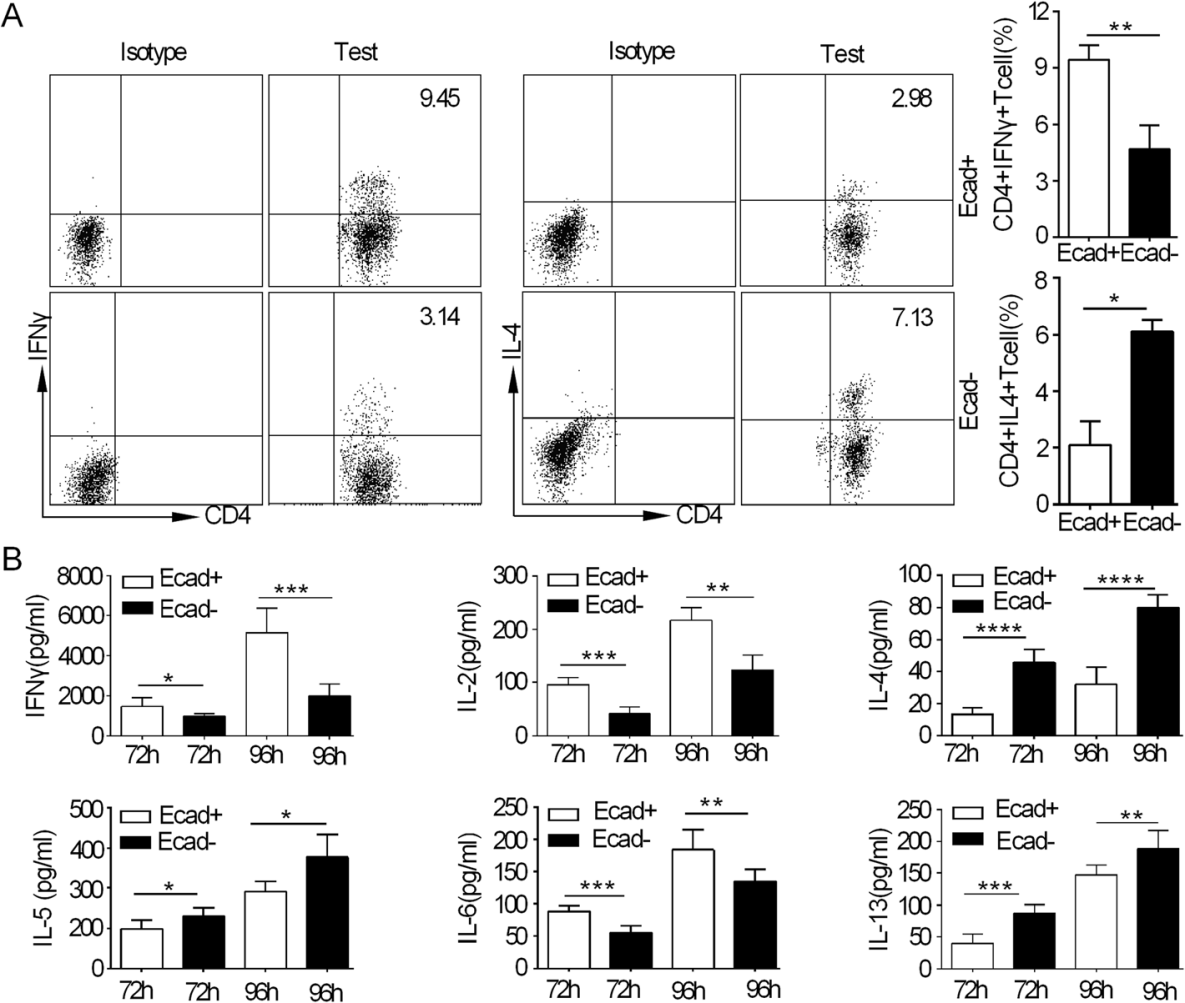
**Relationship between inflammatory E-cadherin + DCs and Th1 and Th2 cells. (A)** The polarisation of Th1 cells with the percentage of CD4 + IFN-γ + T cells and the polarisation of Th2 cells with percentage of CD4 + IL-4+ T cells. Percentage of IFN-γ + CD4+ cells and IL-4 + CD4+ cells from the E-cadherin + DCs and E-cadherin- DCs groups, according to the gating of CD4+ T cells. **(B)** Secretion of cytokines from Th1 and Th2 cells. Cytokine secretion was measured in the supernatant using the FlowCytomix kit (eBioscience). The supernatant was harvested at 72 h and 96 h and then analysed for Th1 (IFN-γ) and Th2 (IL-4, IL-5, IL-6, IL-13). Each experiment was performed in triplicate. The asterisk indicates a significant difference between the two test groups, as analysed by Student’s t-test. (*P < 0.05, **P < 0.01, ***P < 0.001, ****P < 0.0001).

Inflammatory E-cadherin + BM-DCs isolated from the CD4 + CD45RB^high^ T cell colitis model promote the Th17 response, whereas the Th1 response is not significantly altered from that in response to E-cadherin- BM-DCs [3]. To investigate the effects of CD40-mediated E-cadherin + DCs isolated from the spleens of anti-CD40 Rag1 KO mice on the Th1 and Th2 responses in the tumour microenvironment, naive CD4+ T cells were incubated for 96 h with E-cadherin + DCs or E-cadherin- DCs, both of which had been pre-incubated with 5 μg/ml CEA421-435 peptide for 24 h. We observed that the E-cadherin + DCs increased the number of IFN-γ + CD4+ T cells and decreased the number of IL-4 + CD4+ T cells (Figure 2A), whereas extracellularly, IFN-γ and IL-2 secretion was greater than that of the E-cadherin- DC group. However, the secretion of IL-4, IL-5 and IL-13 decreased when compared with that of E-cadherin- DCs. Interestingly, in the E-cadherin + DCs, the secretion of IL-6 was significantly greater than that in the E-cadherin- DCs (Figure 2B). These data suggest that CD40-mediated inflammatory E-cadherin + DCs are able to promote the Th1 response and inhibit the Th2 response in tumour immunity when carrying the CEA peptide.

### E-cadherin + DCs promote the Th17 response and decrease the percentage of Treg cells

**Figure 3:**
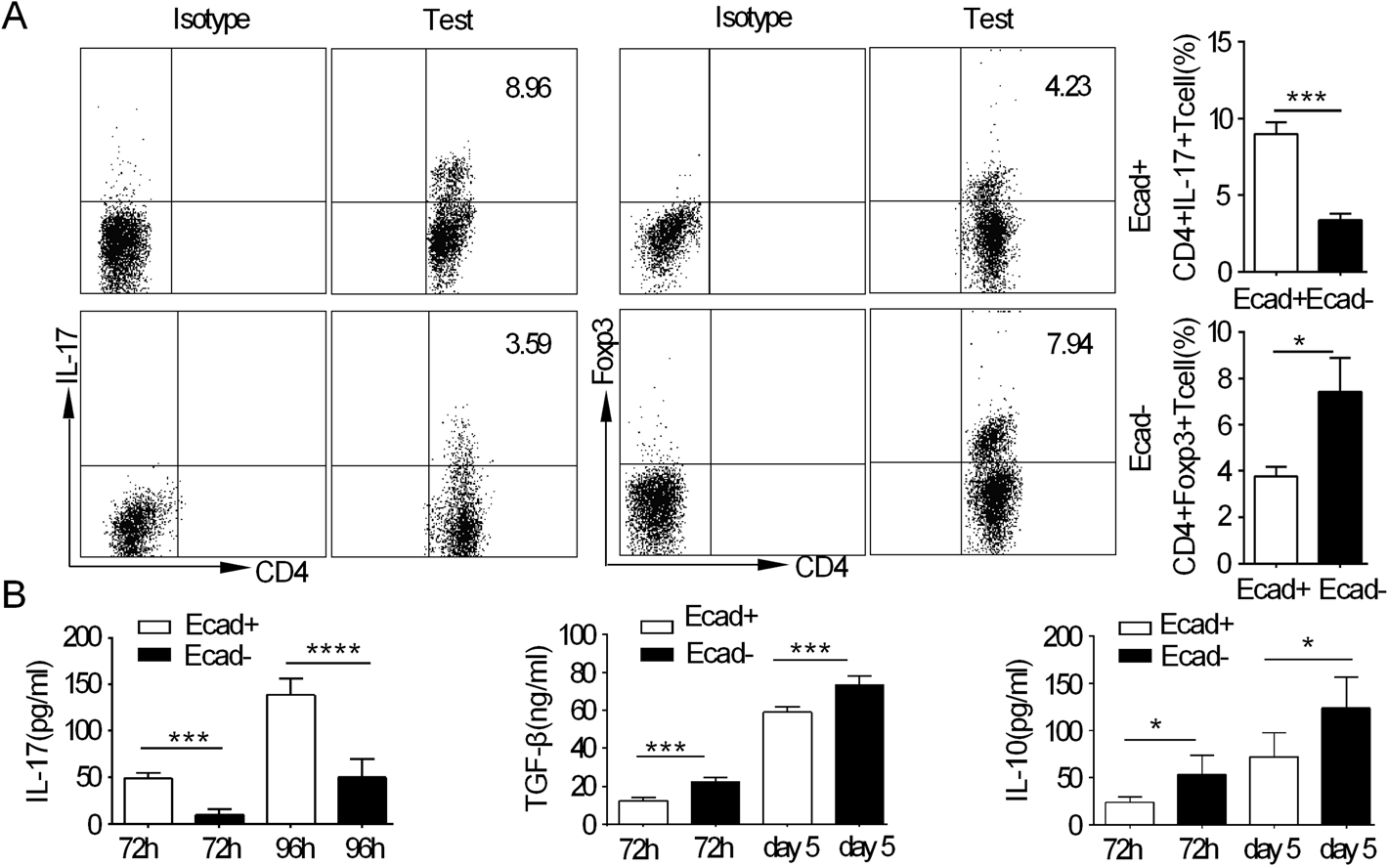
**The effects of inflammatory E-cadherin + DCs on Th17 and Treg cells. (A)** The polarisation of Th17 cells with the percentage of CD4 + IL-17+ T cells and polarisation of Treg cells with the percentage of CD4 + Foxp3+ T cells. Percentage of Foxp3 + CD4+ cells and IL-17 + CD4+ cells from the E-cadherin + DCs and E-cadherin- DCs groups, according to the gating of CD4 + T cells. **(B)** Cytokine secretion was measured in the supernatant using the FlowCytomix kit (eBioscience). The supernatant harvested at 72 h and 96 h was used to assess IL-17 secretion, and the supernatant harvested at 72 h and on day 5 was used to assess IL-10 and TGF-β secretion. Each experiment was performed in triplicate. An asterisk indicates a significant difference between the two test groups, as analysed by Student’s t-test (*P < 0.05, ***P < 0.001).

The differentiation of Th17 cells was enhanced by E-cadherin + DCs in vivo. However, no differences were observed in the frequency of Treg cells compared with E-cadherin- BM-DCs [3]. In light of these findings, we examined the effects of the CD40-mediated E-cadherin + DCs on Th17 and Treg cells. The sorted naive CD4+ T cells were incubated with the CEA421-435 peptide. We showed that the addition of E-cadherin + DCs led to a profound upregulation of Th17 polarisation and reduced the polarisation of Treg cells. Changes in the same functions of Th17 cells and Treg cells were weak in the E-cadherin- DC controls (Figure 3A). We then analysed the secretion of IL-17, IL-10 and TGF-β in the presence or absence of anti-CD40 spleen-derived E-cadherin + DCs. Regarding the secretion of Th17 and Treg cell cytokines, a similar difference was observed between the E-cadherin + DCs and E-cadherin- DCs. Following the addition of E-cadherin + DCs, the secretion of IL-17 was greater than that of the E-cadherin- DC controls; nevertheless, the secretion of IL-10 and TGF-β was decreased compared with the E-cadherin- DCs (Figure 3B). We concluded that CD40-mediated E-cadherin DCs are potent inducers of Th17 cell-mediated responses and suppressors of Treg cell differentiation when carrying the CEA peptide in vitro.

### E-cadherin + DCs enhance the T cell response in orthotopic Lewis lung cancer model

**Figure 4:**
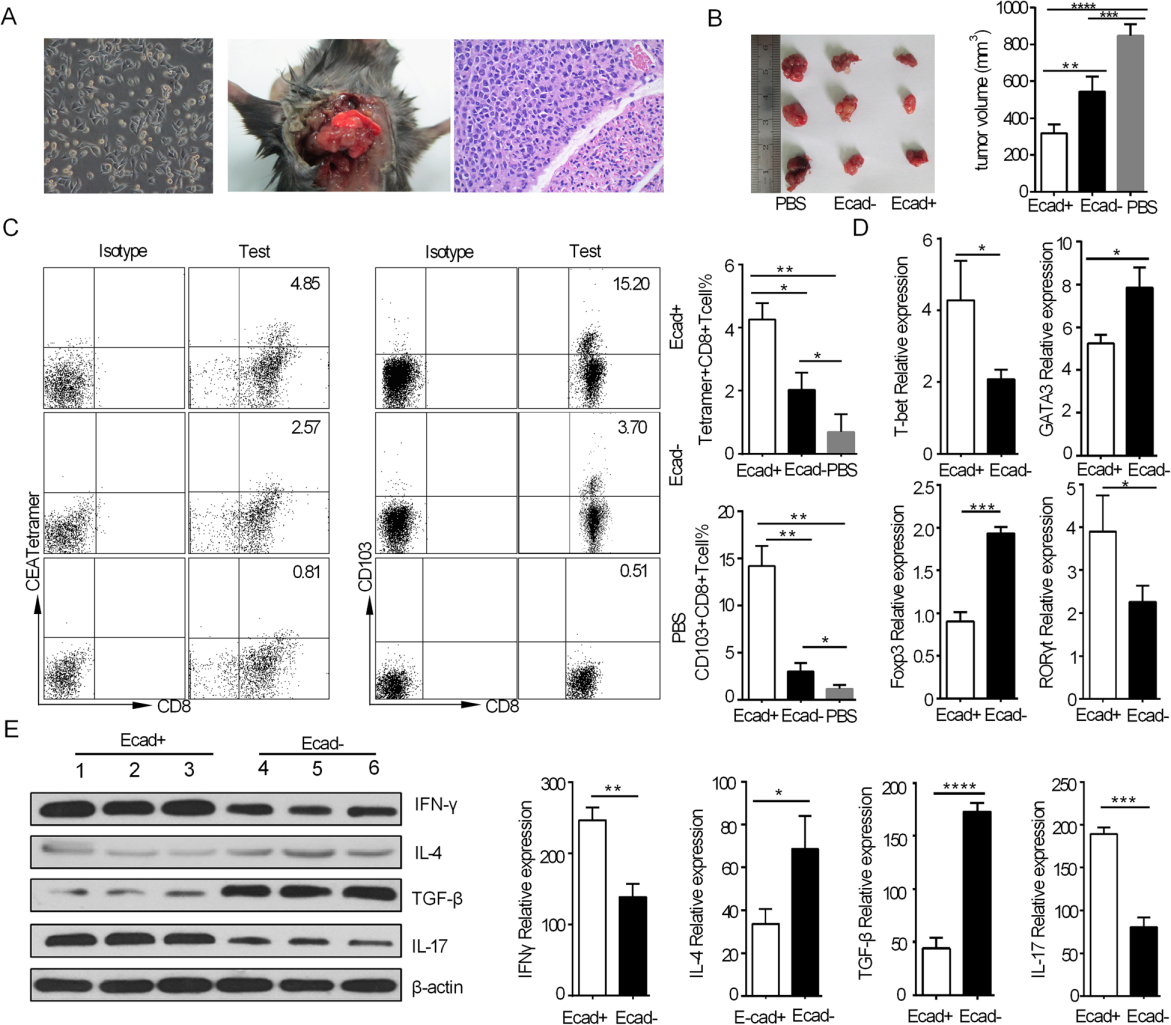
**The effect of inflammatory E-cadherin + DCs on CD4 helper T cell, CEA-specific CD8+ T cell and CD103 + CD8+ T cell responses in an orthotopic lung tumour model. (A)** The microscopic image of a Lewis cell, an HE-stained image and the established orthotopic lung tumour model at day 7 (at day 7, 2 tumour-bearing mice were sacrificed, and the tumour tissues were stained with HE). **(B)** The variations of orthotopic lung tumour volume (day 28) in response to injection of E-cadherin + DCs, E-cadherin- DCs and PBS (each group, n = 5) and representative image of the tumours formed. **(C)** The percentage of CEA tetramer + CD8+ T cells and CD103 + CD8+ T cells, as gated on CD8+ T cells, in the E-cadherin + DCs, E-cadherin- DCs and PBS groups. The tumour-bearing mice had been injected with E-cadherin + DCs or E-cadherin- DCs and sacrificed at day 28. Tumour tissues (each group n = 3) were analysed by qPCR and western blotting to assess Th1, Th2, Th17 and Treg cells. **(D)** Relative gene expression of T-bet, GATA3, Foxp3 and RORγt by PCR. **(E)** Relative protein expression of IFN-γ, IL-4, TGF-β and IL-17 by western blotting. Each experiment was performed in triplicate. An asterisk indicates a significant difference between the two test groups, as analysed by Student’s t-test (*P < 0.05, **P < 0.01, ***P < 0.001 and ****P < 0.0001).

We have shown that CD40-mediated inflammatory E-cadherin + DCs enhance T cell responses in tumour immunity in vitro. However, T cell responses are typically inhibited in tumour immunity, especially in effector CD4+ T cells (Th1, Th2 and Th17 responses). Inflammatory DCs have an excellent ability to activate naive T cells. To assay the effect of inflammatory E-cadherin + DCs on the T cell response and antitumour activity in vivo, we transferred 5 × 10^5^ E-cadherin + DCs loaded with the CEA526-533 peptide into an orthotopic lung tumour model by tail-vein injection on days 7, 14, and 21 following tumour establishment. On day 28, five of the tumour-bearing mice were sacrificed, and the measured tumour volumes of those mice injected with E-cadherin + DCs were reduced relative to mice that received E-cadherin- DCs or the PBS control (Figure 4A-B). Interestingly, upon staining the tetramers of PE-CEA526-533/H-2Db in the spleen lymphocytes of tumour-bearing mice, we found that the percentage of CD8+ CEA526-533 tetramer + T cells was greater than that in mice that received E-cadherin- DCs and the PBS control mice (Figure 4C). We has observed the similar variation of CD8+ CEA526-533 tetramer + T cells on day14 and day 21 (Additional file 1: Figure S5). CD103 + CD8+ T cells have been shown to have an effective role in inhibiting breast cancer and glioma progression [12,13]. However, the effects of these DCs on lung CD103 + CD8+ T cells are unknown. In the lung cells of tumour-bearing mice injected with E-cadherin + DCs, we showed that the percentage of CD8+ T cells expressing CD103 was approximately 15.2%, whereas those of mice injected with E-cadherin- DCs or PBS were approximately 3.7% and 0.51%, respectively (Figure 4C), suggesting that CD40-mediated inflammatory E-cadherin + DCs enhance CEA-specific, CD8+ T cell responses and the expression of CD103 by CD8+ T cells in the lung tumour immune microenvironment.

To investigate the effect of CD40-mediated E-cadherin + DCs on CD4+ T lymphocytes of lung tumours, tumour tissue was harvested for qRT-PCR and western blotting. In this tumour immunity microenvironment model, with E-cadherin + DC treatment carrying the CEA526-533 antigen peptide, the transcription of T-bet and RORγt was enhanced compared with mice that received E-cadherin- DCs and the PBS control mice, whereas FOXP3 transcription was reduced (Figure 4D). The protein levels of IFN-γ and IL-17 were increased in the E-cadherin + DC group, whereas TGF-β was decreased. The transcription of GATA3 and the protein level of IL-4 were reduced in the tumour tissue exposed to E-cadherin + DCs relative to that exposed to E-cadherin- DCs (Figure 4D-E).

### The effects of E-cadherin + DCs on antitumour activity in Lewis lung cancer model

**Figure 5:**
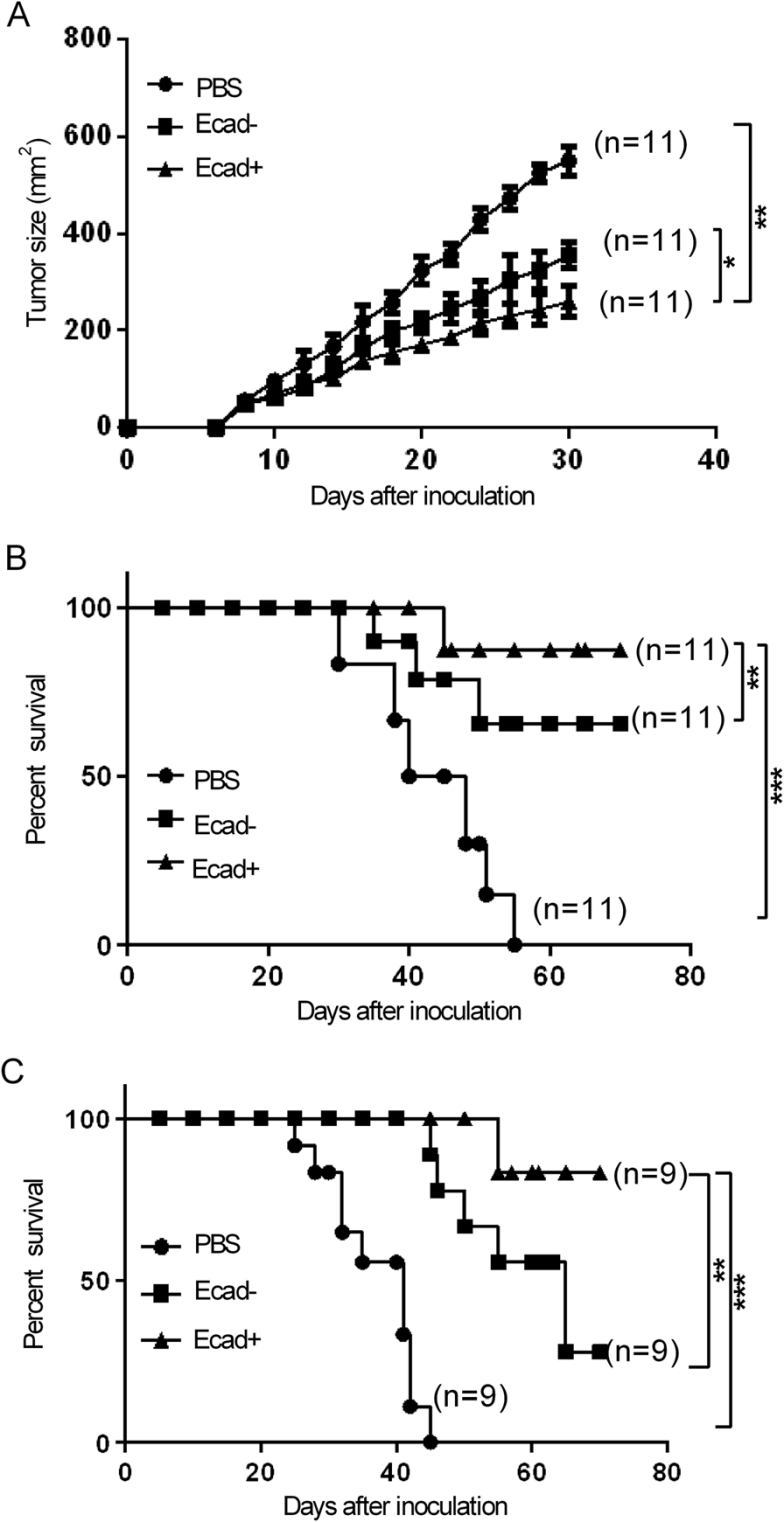
**Therapeutic efficacy of E-cadherin + DCs on lung tumour-bearing mice. (A)** Changes in the tumour size in subcutaneous lung tumour-bearing mice. The tumour diameters (width*length) were measured using callipers every 2–3 days for the subcutaneous tumour model after E-cadherin + DCs were injected (each group n = 11). The mean size of the subcutaneous tumours of the mice that developed tumours in each group after tumour challenge. Three individual mice per group were analysed. In the E-cadherin + DC group, tumour volume decreased significantly compared with the E-cadherin- DC group. In the E-cadherin + DC and E-cadherin- DC groups, tumour volume increased slower than in the PBS group. **(B)** Survival curves of the subcutaneous tumour-bearing mice (each group n = 11). The E-cadherin + DC groups survived longer than the E-cadherin- DC groups, and both the E-cadherin + DC and E-cadherin- DC group survived longer than the PBS group. **(C)** Survival curves of the orthotopic lung tumour-bearing mice (each group n = 9). The E-cadherin + DC group survived longer than the E-cadherin- DC group, and both the E-cadherin + DC and E-cadherin- DC groups survived longer than the PBS group. Each experiment was performed in triplicate. The data were analysed by Student’s t-test (*P < 0.05, **P < 0.01, ***P < 0.001).

To further determine whether E-cadherin + DCs enhanced the antitumour activity and prolonged the survival time of tumour-bearing mice, we monitored tumour growth and the survival time of the subcutaneous model, as well as the survival time for the orthotopic lung tumour model (Figure 5A-C). Following transfer of E-cadherin + DCs loaded with the CEA526-533 peptide, the subcutaneous tumour volume decreased significantly compared with the E-cadherin- DC group. The E-cadherin + DCs prolonged the survival time of the subcutaneous and orthotopic lung tumour-bearing mice. However, no mice survived in the PBS-vaccinated group.

## Discussion

A subset of DCs not found in the steady state occurs as a consequence of inflammation or antibody-mediated sterile inflammation. These DCs have been termed inflammatory DCs. Inflammatory monocytes are the main precursors of inflammatory DCs [14]. These precursors, such as Gr1 + CD115+ inflammatory monocytes, can differentiate into E-cadherin + DCs through GM-CSF mediation [3]. Although a few DCs express E-cadherin in normal mice, the inflammatory E-cadherin + DCs detected in the lungs of the lung tumour model are not detected. Here, we demonstrated that E-cadherin + DCs accumulated in the lung and spleen during CD40 mAb-mediated innate immunity, indicating that the provisions of the CD40 signalling pathway are sufficient to drive the accumulation of inflammatory DCs expressing E-cadherin.

Monocyte-derived inflammatory DCs play an early role in adaptive immunity [15]. However, these cells have not been shown to prime naive T cells in vivo, and their functions in tumour immunity in vitro and in vivo are unknown. The differences in the activities of DC subsets on naive T cells depend on the phenotype of the DCs. CD8α + DCs could play a role in tolerance induction and restrict the immune response, whereas CD8α- DCs could be stimulatory to the T cell response [16]. CD40-mediated inflammatory E-cadherin + DCs do not express CD8α and CD4. Surprisingly, Pulendran et al. and Maldonado-Lopez et al. demonstrated that CD8α + DCs lead to Th1 differentiation and that CD8α- DCs induce a Th2-type response [17,18]. Although E-cadherin + and E-cadherin- BM-DCs failed to promote IFN-γCD4^+^ T cell generation, due to increased overall cell numbers, E-cadherin+, but not E-cadherin-, BM-DCs enhanced the Th1 and Th17 responses. E-cadherin + BM-DCs derive from inflammatory Gr1+ monocytes and express Gr1 [3]. Blood-derived Gr1^+^ inflammatory DCs can induce a Th1 response to infection in vivo [19]. Here, CD40-mediated inflammatory E-cadherin + DCs promoted the Th1 response, whereas Th2 responses were decreased when these DCs were carrying the CEA antigen peptide. As IFNγ and IL-12 dominate, the enhanced Th1 response can inhibit Th2 cell development through a reduction in IL-4 expression [20]. The Th1 response promotes host responses to tumours because IFN-γ and IL-2 can prime the CD8+ T cell response, thereby protecting the host by monitoring against tumour development [21-23]. Nevertheless, the effect of the Th2 response on the tumour is controversial. In the 1990s, IL-4 was identified as a potent anti-tumour factor [24]. Recently, the Th2 response has been considered a factor promoting tumour growth that affects CD8^+^ T cells. IL-4 was found to stimulate CD8^+^ T cells to differentiate into non-lytic CD4^−^CD8^−^ T cells and to reduce the susceptibility of human CD8^+^ T cells to activate induced cell death [25].

The effect of inflammatory E-cadherin + DCs to the blance of Th17/Treg is unknown in tumour immune microenvironment. As is well-known that Treg cells contribute to the progression of cancer by suppressing antitumor immunity [26,27]. Even though it is protective factors in colitis, with suppressive functions through IL-10 and FOXP3 [28]. Inflammatory E-cadherin + DCs from the CD4+ CD45RB^high^ T cell model demonstrate a potent ability to induce T cell-mediated colitis with Th17 responses; the effect on Treg cells was not significant with E-cadherin- DCs [3]. However, we found that CD40-mediated E-cadherin + DCs enriched the Th17 response and significantly inhibited the Treg response when the DCs were carrying the CEA peptide. Treg-mediated suppression is a crucial component of CD8^+^ T cell repopulation [29]. Th17 cells are related to IL-17 and mediated by Th17-stimulated CD8^+^ T cells in the induction of preventive and therapeutic antitumour immunity [30]. However, IL-17 and IL-23 have been found to drive tumour growth in colorectal cancer with Th17-mediated intestinal inflammation [31]. With Treg cell development impaired by E-cadherin^+^ DCs and the increased number of Th17 cells, CEA-specific CD8+ T cell responses are enhanced in the in vivo lung tumour model. Tumour volume decreased, and the survival time of tumour-bearing mice was prolonged significantly following the injection of CD40-mediated inflammatory E-cadherin^+^ DCs compared with injection of E-cadherin^−^ DCs. These results may be relevant to the comprehensive effects of Th1 and Th17 on the proliferation of tumour antigen-specific CD8+ T cell responses. The promotion of tumour growth by CD4+ helper T cells and CD8+ T cells is subdued with the intervention of CD40-mediated inflammatory E-cadherin + DCs. In particular, as the CD40 signalling pathway is activated, the expression of CD40 in the E-cadherin^+^ DCs is upregulated, and naive CD4^+^ and CD8^+^ T cells are primed because helper T cells and the generation of CTLs by cross-priming are mediated by signalling through CD40 on the antigen-presenting cell [32].

Interestingly, the results of transferring CD40-mediated E-cadherin + DCs into the Lewis lung tumour model suggest that the development of helper T cells in vivo is similar to that in the in vitro experiments. Furthermore, the tumour-specific CD8+ T cell responses were significantly enhanced following the addition of E-cadherin + DCs carrying CEA in vivo. Additionally, when E-cadherin + DCs were cultured with naïve CD8+ T cells, the production of IFN-γ was greater than when E -cadherin^−^ DCs were used (Additional file 1: Figure S3). In the Th1 response, CD8 cytotoxic-T cell responses increased, Treg responses decreased, and the tumour volume was decreased. CD40-mediated inflammatory E-cadherin + DCs therefore possess an excellent antitumour ability via enhancement of the anti-tumour T cell response and suppression of Treg cell development. Recently, CD103 + CD8+ T cells have been shown to be effective in inhibiting breast cancer and glioma progression. These CD103^+^CD8^+^ T cells can also upregulate CD8+ T cell cytotoxic mediators when in contact with their specific antigen. CD103^+^CD8^+^ T cells also contribute to protecting the human lung against viral infection by producing IFN-γ and other Th1 cytokines, such as IL-2. In addition, these cells have an effector or memory phenotype [33]. In our vivo experiments, we demonstrated that E-cadherin + DCs are able to enhance the development and accumulation of CD103^+^CD8^+^ T cells. The ligand for CD103 is E-cadherin; thus, E-cadherin-expressing DCs are more likely to be recognised and combined by CD103^+^CD8^+^ T cells during antigen peptide capture, thereby activating these CD8+ T cells.

## Conclusions

Taken together, these results strongly suggest that Anti-CD40-induced inflammatory E-cadherin + DCs promote T cell responses and antitumour activity in murine Lewis lung carcinoma. It is unknown why inflammatory E-cadherin^+^ DCs accumulate in the presence of CD40 signalling pathway activation only in innate immunity. A further understanding of the mechanisms of CD40 signalling pathway-mediated inflammatory E-cadherin + DC differentiation in innate immunity and the tumour microenvironment may provide novel therapeutic strategies and insight into the pathogenesis of NSCLC.

## Additional file

Additional file 1: **Supplementary material.** **Figure S1.** Purities of the pre-sorted and after-sorted CD40-mediated inflammatory E-cadherin^+^ DCs. **Figure S2.** Purities and phenotypes of pre-sorted and after-sorted naive CD4^+^ T cells and naïve CD8^+^ T cells. **Figure S3.** The secretion of IFN-γof E-cadherin+DCs group and E-cadherin-DCs group that had cultured with naive CD8+T cells respectively. **Figure S4.** Purified agonist CD40 antibody and established anti-CD40 model. **Figure S5.** The effect of inflammatory E-cadherin+ DCs on CEA-specific CD8+ T cell responses in an orthotopic lung tumour model [34,35].
