# Supplementary material for: Anti-CD40-induced inflammatory E-cadherin+ dendritic cells enhance T cell responses and antitumour immunity in murine Lewis lung carcinoma
Source: J Exp Clin Cancer Res. 2015 Feb 5;34(1):11. doi: 10.1186/s13046-015-0126-9 (PMC4323023; doi:10.1186/s13046-015-0126-9)
Supplement: Supplementary file 1 — Supplementary material. Figure S1. Purities of the pre-sorted and after-sorted CD40-mediated inflammatory E-cadherin+ DCs. Figure S2. Purities and phenotypes of pre-sorted and after-sorted naive CD4+ T cells and naïve CD8+ T cells. Figure S3. The secretion of IFN-γof E-cadherin+DCs group and E-cadherin-DCs group that had cultured with naive CD8+T cells respectively. Figure S4. Purified agonist CD40 antibody and established anti-CD40 model. Figure S5. The effect of inflammatory E-cadherin+ DCs on CEA-specific CD8+ T cell responses in an orthotopic lung tumour model [34,35]. [file 13046_2015_126_MOESM1_ESM.doc]

**Supplementary material**

**Reagents and antibodies**

The following anti-mouse monoclonal antibodies were used for FACS analysis: PE-E-cadherin (BD Biosciences, Cat NO. 562526), APC-cy7-CD11c (BD Biosciences, Cat NO. 561241), APC-CD4 (BD Biosciences, Cat NO. 553051), FITC-CD103 (BD Biosciences, Cat NO. 557494) and 7-AAD (BD Biosciences, Cat NO. 559925). The following antibodies and reagents were used for T cell culture: anti-mouse CD3e（eBioscience，Clone 145-2C11），anti-mouse CD28（eBioscience，Clone 37.51 ），and recombinant mouse IL-2 (R&D systems，Cat NO. 402-ML). For naive CD4+ and CD8+ T cell purification, FACS was performed with antibodies to APC-CD4，FITC-CD8, percp-cy5.5-CD44 and PE-CD62L (eBioscience). For cytokine analysis from cell suspensions, the mouse IFN-γ ELISA Kit（R&D systems，Cat NO. MIF00），mouse Th1/Th2/Th17/Th22 13-plex kit （eBioscience，Cat NO. BMS822FF），mouse TGF-β1 simplex kit （eBioscience，Cat NO. BMS8608FF）and mouse basic kit (eBioscience, Cat NO. BMS8440FF) were utilised. For CD4+ T cell polarization analysis, the mouse Th1/Th2/Th17 phenotyping kit (BD Biosciences, Cat NO. 560758), mouse Th1/Treg phenotyping kit (BD Biosciences, Cat NO. 560767) were used. For confocal microscopy, the anti-CD11c antibody (abcam, Cat NO. ab33483), anti-E-cadherin antibody (BD Biosciences, Cat NO. 610181), goat anti-mouse antibody, goat anti-Armenian hamster IgG-FITC (Santa Cruz Biotech, Cat NO. sc2446) and goat anti-rabbit IgG-PE (Santa Cruz Biotech，Cat NO. sc3739) were used. The CEA526-533H-2Db peptide (EAQNTTYL, purity>95%) and CEA421-435H-2b peptide (SPSYTYYRPGVNLSL, purity>95%) were synthesised by Invitrogen Corporation; all peptides were stored at -80°C and subpackaged at -20°C for short-term storage. The CEA-526-533/H-2Db tetramer and HIV-gag390-398/H-2Db tetramer were purchased from Beckman Coulter (Fullerton，CA). FGK45 cells were cultured in Protein-Free Hybridoma Medium II (PFHM-II, Invitrogen). Antibodies for western blots were from the following R&D Systems：IFN-γantibody，mouse IL-4 MAb，TGF-beta 1 MAb and mouse IL-17 MAb.

**Cell culture**

The Lewis lung carcinoma (LLC) cell line was purchased from the Chinese Academy Cell Resource Centre at the Shanghai Institutes for Biological Sciences. The cells were cultured in Dulbecco’s Modified Eagle Medium (DMEM, GIBCO, Invitrogen) and supplemented with 10% foetal bovine serum (FBS, GIBCO, Invitrogen), streptomycin (100 µg/ml) and penicillin (100 U/ml). The cells were maintained in 10-cm cell culture dishes at 37°C in an incubator containing 5% CO_2_.FGK45 hybridoma cells were obtained as a gift from Prof. Antonius Rolink (Basel Institute for Immunology, Basel, Switzerland) and cultured in Protein-Free Hybridoma Medium II .Cell line authentication was performed according to UKCCCR Guidelines every 2-3 months，including mycoplasma test by PCR and measurement of cell proliferation by counting.

**Tumour cells**

The Lewis lung carcinoma (LLC) cell line was purchased from the Chinese Academy Cell Resource Centre at the Shanghai Institutes for Biological Sciences. The cells were cultured in Dulbecco’s Modified Eagle Medium (DMEM, GIBCO, Invitrogen) and supplemented with 10% foetal bovine serum (FBS, GIBCO, Invitrogen), streptomycin (100 µg/ml) and penicillin (100 U/ml). The cells were maintained in 10-cm cell culture dishes at 37°C in an incubator containing 5% CO_2_.

**lung tumour model**

LLC cells were mixed at a 1:1 dilution with Matrigel (BD Biosciences, Cat NO. 356234). The lungs of C57BL/6 mice were injected with the LLC cells（5*106/50 µl per mouse）as previously described【34】. For establishing subcutaneous tumor model, 2.0 *106 Lewis cells in 100 μL of PBS were implanted subcutaneously in the midflank of C57BL/6 mice on day 0.At day 7, tumour-bearing mice were euthanised, and the tumours were measured for length（a），width（b） and height(c); tumour volumes were calculated using 0.52*ab2.【35】. The diameters of the tumors (width * length) were measured by calipers every 2–3d with subcutaneous tumor model.

**Purified CD40 agonist antibody**

The agonistic anti-mouse CD40 monoclonal antibody was purified from FGK45 cell supernatant. Following culture in Protein-Free Hybridoma Medium II (PFHM-II, Life Technologies) andβmercaptoethanol（1μg/ml，Sigma）, 3000 ml of FGK45 cell supernatant was used to purify the anti-mouse CD40 monoclonal antibody using a Protein G column, resulting in 19.2 mg of anti-mouse CD40 antibody, which contained <0.1 EU endotoxin per milligram of protein.(Figure S4A)

**Anti-CD40 model**

Rag1-/- mice received intraperitoneal injections containing 200 µg of agonistic FGK45 anti-mouse CD40 monoclonal antibody. Mice were weighed daily and euthanised at day 7. The lungs and colons of the anti-CD40 mice were fixed with 4% paraformaldehyde and stained with hematoxylin and eosin. (Figure S4B-D)

**Flow cytometry**

Cells（10^6^/tube）were kept on ice throughout the procedure according to the protocol described by BD Biosciences and were incubated for 30 min with optimal concentrations of fluorescent antibodies in the dark. Controls included unstained cells and stained isotype-matched antibodies. For assaying the subsets of T cell differentiation and polarisation, Th1，Th2，Th17 and Treg cells and related cytokines were detected using the corresponding reagent kit and the procedure recommended by the manufacturer. For tetramer staining， cells were prepared from the spleen tissue of lung tumour-bearing mice that had been injected with E-cadherin+ or E-cadherin- DCs; controls were injected with 200 μl PBS alone. Spleen lymphocytes of orthotopic tumour-bearing mice were stained with the anti-CD3e monoclonal antibody （mAb），anti-CD8（mAb）and PE-conjugated CEA_526-533_/H-2D^b^tetramer (Beckman Coulter, Fullerton, CA) negative controls were stained with the PE-conjugated HIV-gag390-398/H-2Db tetramer. The cells were analysed on a BD Biosciences FACSAria^TM^III, and the data were analysed with FlowJo（Tree Star）

**Fluorescent staining and confocal microscopy**

Tissues from the lungs of C57BL/6 mice and rag1 KO mice （i.p. with FGK45 anti-CD40 antibody）and lung tumours from C57BL/6 mice were embedded in OCT（Tissue-Tek®, Torrance, CA）and frozen in liquid nitrogen. Sections (3–5 μm) were fixed in 4% paraformaldehyde at −20°C and blocked with 2% normal goat serum-buffered saline. The tissue sections were stained with an anti-CD11c antibody (1:400 Abcam) and an anti-E-cadherin antibody (1:400 BD Biosciences) overnight at 4°C. After washing 3 times with TBS, sections were incubated with the corresponding secondary antibody: goat anti-mouse antibody, goat anti-Armenian hamster IgG-FITC and goat anti-rabbit IgG-PE for 1 h at room temperature. Finally， sections were mounted in Vectashield with DAPI, followed by analysis on a confocal laser scanning microscope （Fluoview FV1000，Olympus, Tokyo, Japan）.

**Secretion of IFN-γby CD8^+^ T cells**

Supernatants were collected from 96-well plates of DCs cultured with naive CD8+ T cells and stored at -80°C. Secretion of IFN-γfrom the E-cadherin+ and E-cadherin- DC groups were assessed using the IFN-γ ELISA Kit（R&D systems）. All procedures followed the instructions of the manufacturer.

**RNA isolation and quantitative real-time RT-PCR**

Total RNA was purified using TRIzol reagent （Invitrogen，15596026）from lung tumour tissues from tumour-bearing mice. cDNA was synthesised from 1 μg of total RNA using the RevertAid First-Strand cDNA Synthesis Kit (Thermo Scientific, USA). Real-time RT-PCR was performed on a Bio Rad CFX96 sequence detection system (Bio-Rad Laboratories Inc., USA) using Platinum SYBR Green qPCR SuperMix-UDG reagents (Life Technologies, USA). These reactions were incubated at 95°C for 10 min，followed by 40 cycles of 95°C for 15 s, 20 seconds at 60°C, and 20 seconds at 72°C. The following primer pairs were used: T-bet sense 5,一TTCCCATTCCTGTCCTTCAC-3,, antisense 5,一CCTCTGGCTCTCCATCATTC-3,; GATA-3, sense 5,一AGAACCGGCCCCTTATCAA-3,, antisense 5,一AGTTCGCGCAGGATGTCC-3,; Foxp3 sense 5,一CACCTATGCCACCCTTATCC-3,, antisense 5,一CGAACATGCGAGTAAACCAA-3,; RORγt sense 5,一GCGGAGCAGACACACTTACA-3,, antisense 5,一TTGGCAAACTCCACCACATA-3,; and GAPDH sense 5,一TGCAGTGGCAAAGTGGAGATT-3,, antisense 5,一TTGAATTTGCCGTGAGTGGA-3,. Amplification reactions were performed in duplicate，and the T-bet，GAGT3, Foxp3 and RORγt gene expression levels for each sample were normalised to glyceraldehyde-3-phosphate dehydrogenase (GAPDH). Relative gene expression levels were calculated using the 2^- ∆∆ct^ method.

**Western blot**

Total protein was extracted from lung tumour tissue using the Total Protein Extraction Kit (Pierce/Thermo Scientific). The BCA Protein Assay Kit (Pierce) was used to determine the protein concentration. Protein samples were separated on 12% SDS-PAGE and electrotransferred onto nitrocellulose membranes. Membranes were sequentially blocked in TBST including 5% skim milk then incubated with an IFN-γantibody (1:1000，R&D Systems)，mouse IL-4 MAb (1:1000，R&D Systems)，TGF-beta 1 MAb (1:1000，R&D Systems)，mouse IL-17 MAb (1:1000，R&D Systems) and anti-β-actin (1:1000; Abcam) at 4°C overnight. Membranes were washed then incubated with the corresponding HRP-conjugated secondary antibody (1:5000, 37° C, 2 hours). After three final washes， target bands were visualised using an ECL detection system（Thermo Scientific）and semiquantitatively analysed with densitometric methods.

**Supplementary Figure Legends**

**Figure S1** Purities of the pre-sorted and after-sorted CD40-mediated inflammatory E-cadherin^+^ DCs. (A) and (B) After exclusion of the 7-AAD+CD11c-cells from the spleen cell suspension of the anti-CD40 model by a BD FACSAria III flow cytometer, 7-AAD-CD11c^+^ cells were obtained and assayed by FACS. (C) The percentage of E-cadherin expressed on CD11c^high^ cells. (D) and (E) The percentage of CD4 and CD103 expressed on E-cadherin^+^CD11c^high^ cells and E-cadherin^-^CD11c^high^ cells. (F) and (H) The percentage of after-sorted E-cadherin^+^ CD11c cells. (G) and (I) The percentage of CD4 and CD103 expression on after-sorted E-cadherin^+^CD11c^high^ cells and E-cadherin^-^CD11c^high^ cells.

**Figure S2** Purities and phenotypes of pre-sorted and after-sorted naive CD4^+^ T cells and naïve CD8^+^ T cells. Six- to-eight-week-old C57/BL6 mice were sacrificed, and their spleen cells were prepared as a suspension. Then, the cells were stained for CD4, CD8, CD44 and CD62L, submitted to FACS and sorted. (A) The percentage of CD4^+^ T cells and the amounts of CD44 and CD62L expressed on CD4^+^ T cells before sorting. (B) The purity of CD4^+^CD62L^high^CD44^low^ cells from the CD4^+^ T cell population after sorting. (C) The percentage of CD8^+^ T cells and the amounts of CD44 and CD62L expressed on CD8+ T cells before sorting. (D) The purity of CD8+CD62LhighCD44low cells from CD8+ T cells after sorting.

**Figure S3**The secretion of IFN-γof E-cadherin+DCs group and E-cadherin-DCs group that had cultured with naive CD8+T cells respectively. The control was the naive CD8+Tcells cultured with anti-CD3 (1 μg/ml) and anti-CD28 (5 μg/ml)(no DCs)Each experiments performed two times.The asterisk indicates a significant difference between the two test groups, as analyzed by Student’s t-test.（*P<0.05）

**Figure S4** Purified agonist CD40 antibody and established anti-CD40 model.（A）The 5%-15% SDS-PAGE of our purified FGK45 mouse anti-CD40 antibody .（B）The weight change of rag ko mice after injected anti-CD40 mAb 200μg during 7 days.（C）The colon of normal C57/BL6 mice stained with HemateinEosin（HE).（D）The colon of anti-CD40 model mice stained with HE. A lot of granulocyte accumulated under colon mucosa，crypt disordered，and the number of goblet cells reduced.

Figure S5 The effect of inflammatory E-cadherin+ DCs on CEA-specific CD8+ T cell responses in an orthotopic lung tumour model. The percentage of CEA tetramer+CD8+ T cells ，as gated on CD8+ T cells in the E-cadherin+ DCs, E-cadherin- DCs and PBS groups(each group n=3). The tumour-bearing mice had been injected with E-cadherin+ DCs or E-cadherin- DCs and sacrificed at （A）day14 and（B） day21.

**Figure S1**


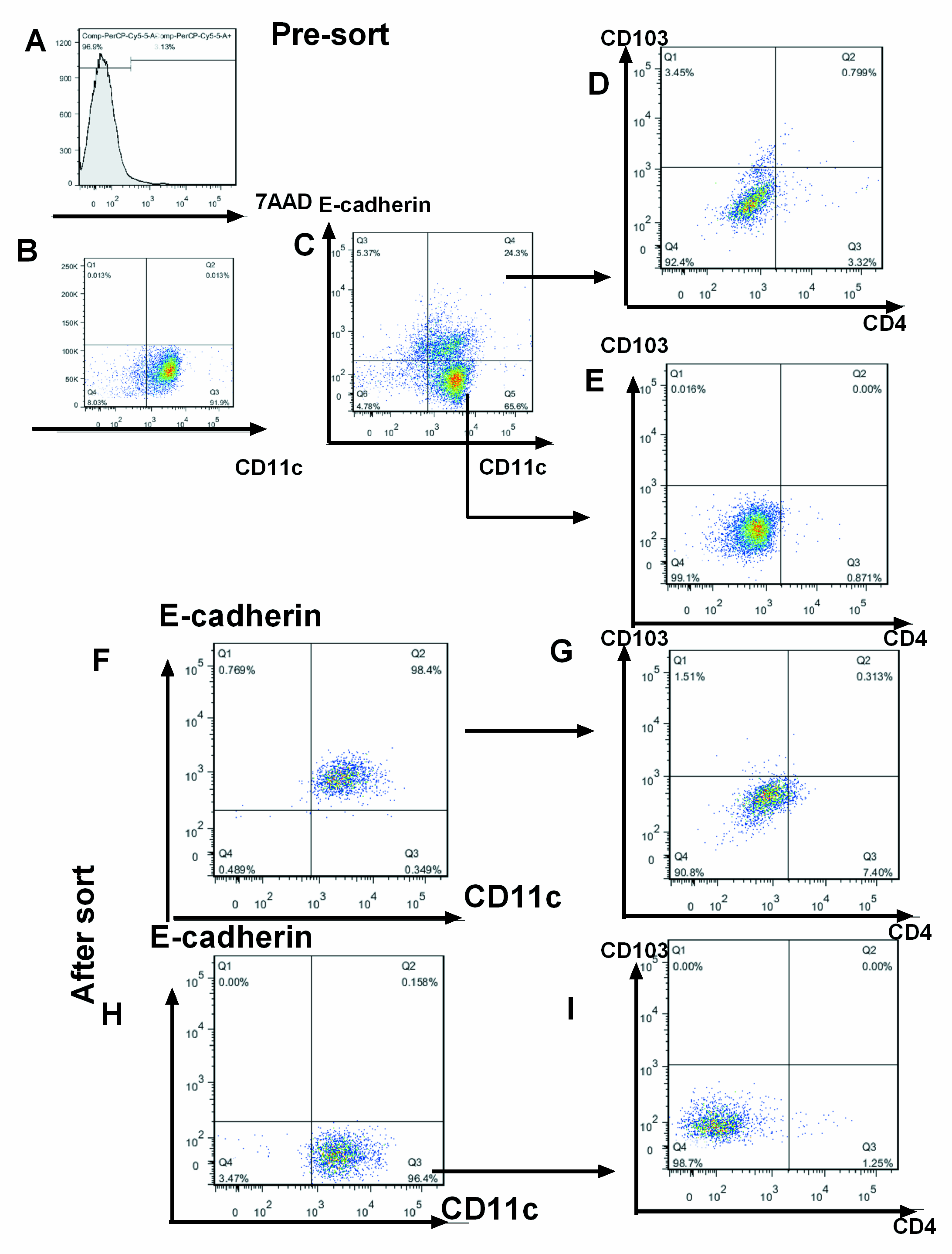


**Figure S2**


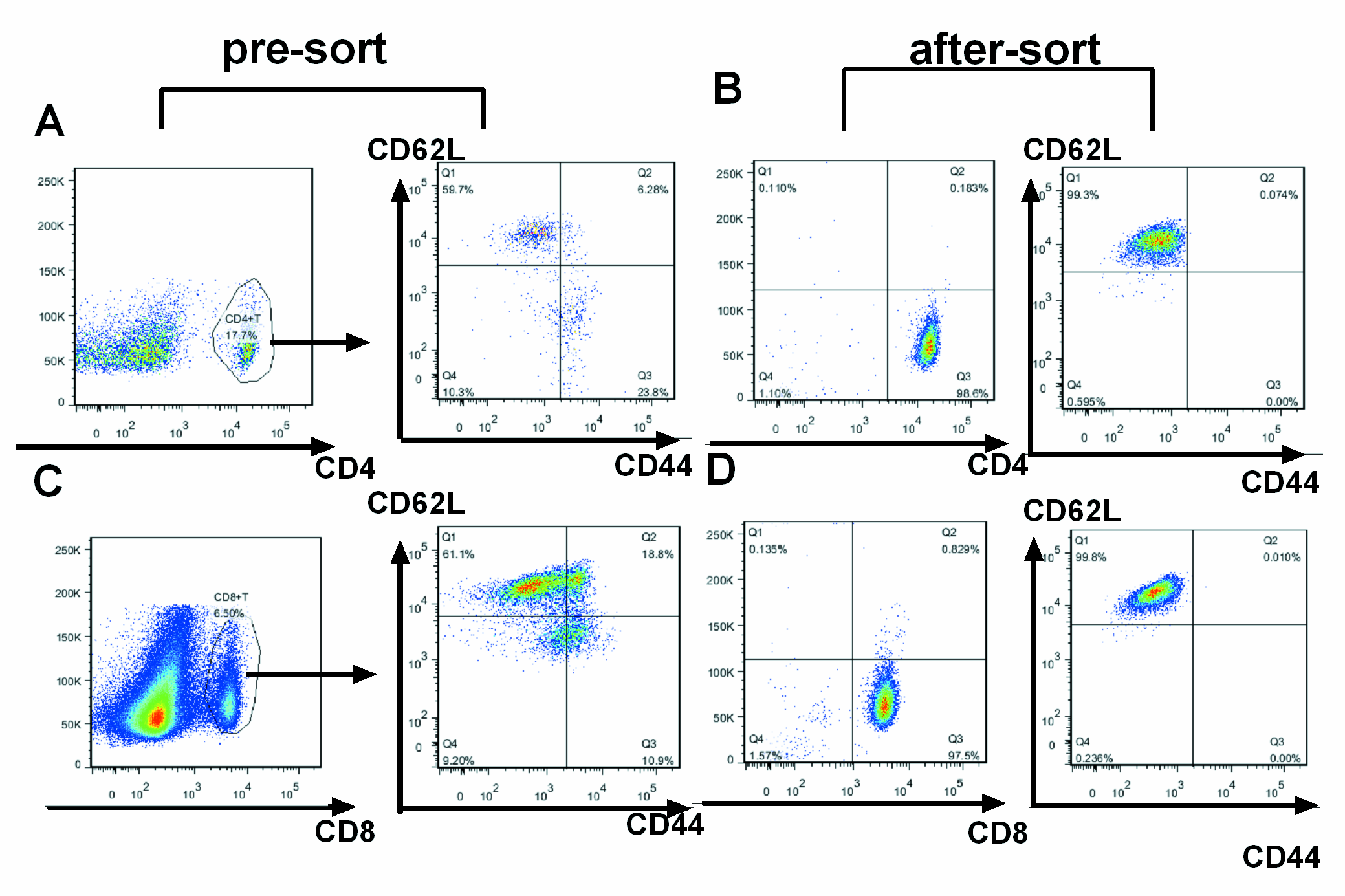


**Figure S 3**

**
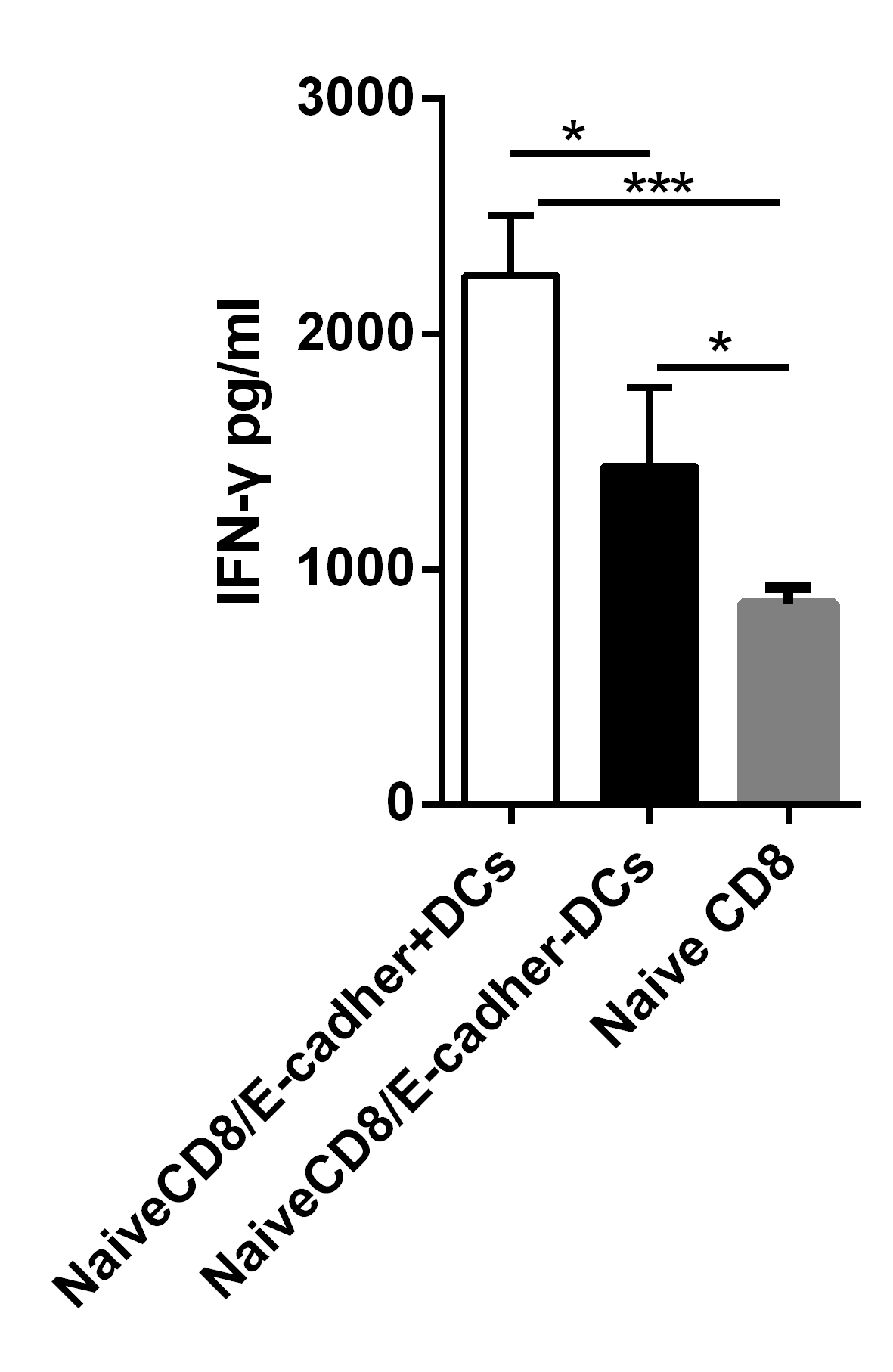
**

**Figure S 4**

**
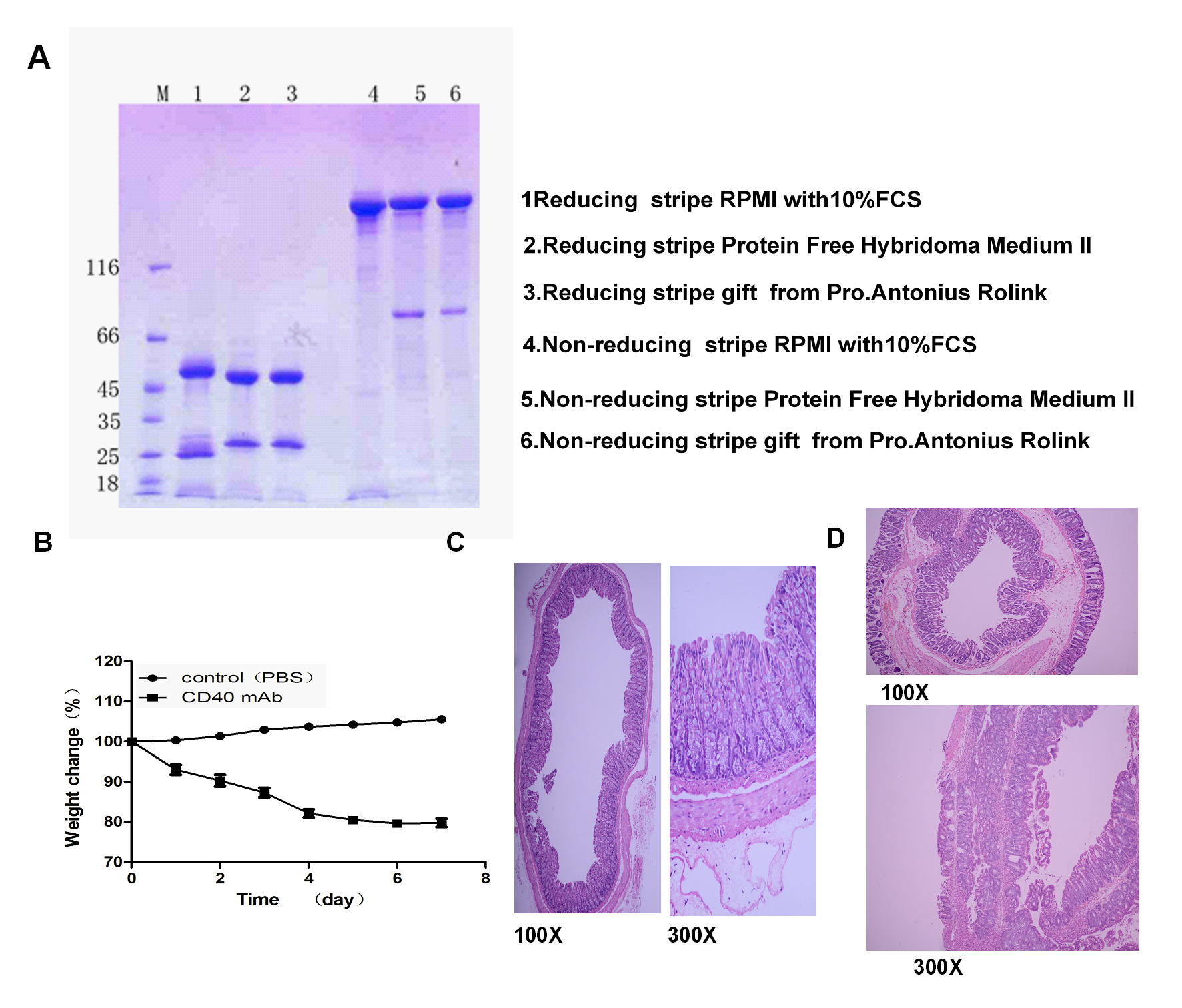
**

**Figure S5**

**
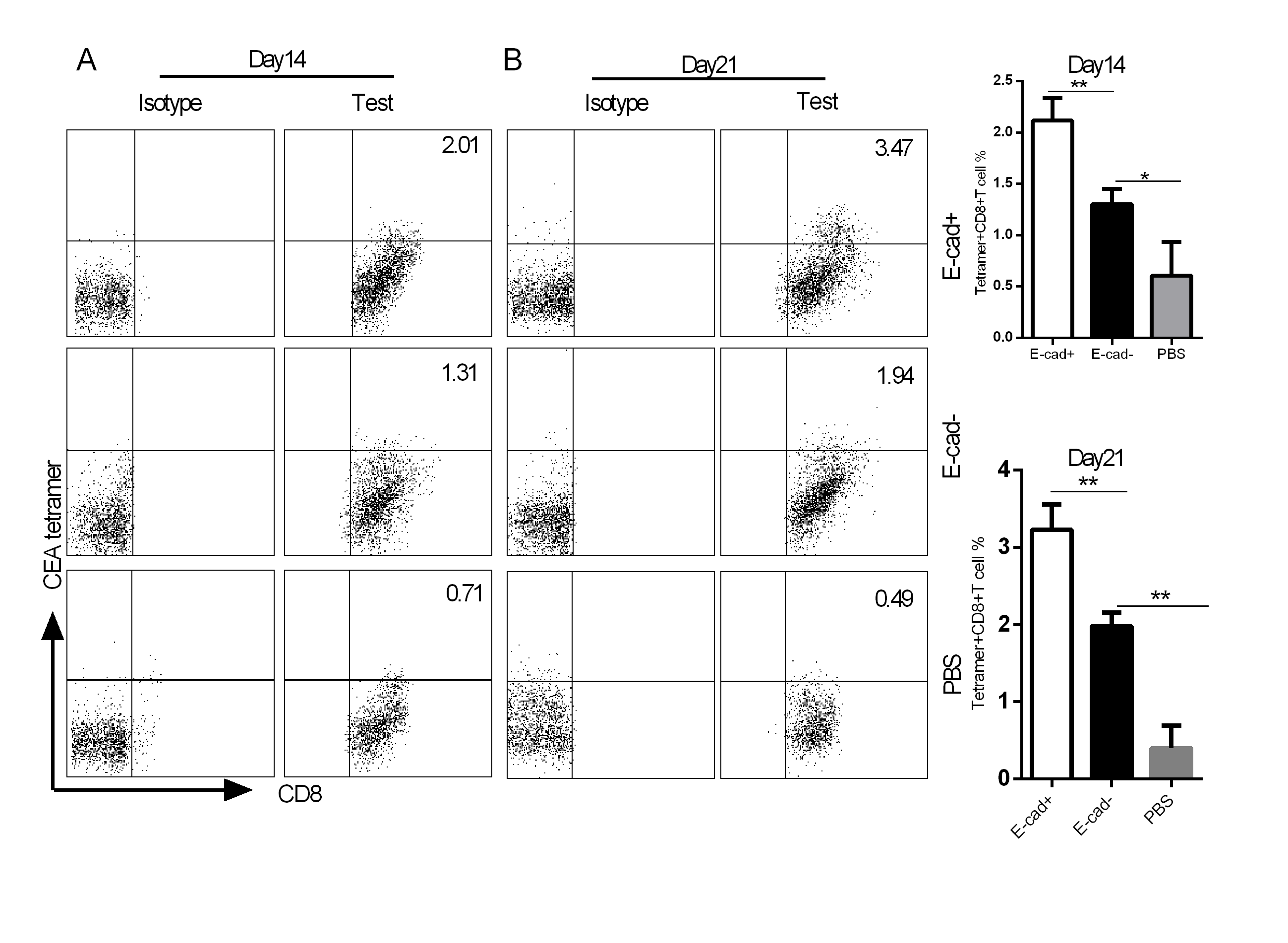
**
